# 16S rRNA gene sequencing dataset describing the diversity and structure of soil bacterial communities across four pesticide-free agroecological cropping systems of arable crops from the CA-SYS experiment between 2018 and 2021

**DOI:** 10.1016/j.dib.2026.112976

**Published:** 2026-06-16

**Authors:** Elizaveta Klockenbring, Julie Aubert, Jérémie Béguet, Stéphane Cordeau, Violaine Deytieux, Céline Faivre, Rodolphe Hugard, Nicolas Jouvin, Samuel Mondy, Brice Mosa, Aymé Spor

**Affiliations:** a Université Bourgogne Europe, Institut Agro, INRAE, UMR Agroécologie 21000 Dijon, France; b Université Paris-Saclay, AgroParisTech, INRAE, UMR MIA Paris-Saclay 91120 Palaiseau, France; c UE 115 Domaine Expérimental d'Époisses, INRAE 21110 Bretenière, France

**Keywords:** Agroecology, Soil microbiology, Metabarcoding, Microbial community, Bioinformatics

## Abstract

A dataset describing soil bacterial community diversity and structure was generated from the CA-SYS experimental platform (INRAE, France), a long-term research facility designed to evaluate agroecological practices under contrasting soil management without pesticide use. Soil samples were collected from 42 experimental plots (4 subplots per plot) across four cropping systems, including no-till and tilled systems with or without nitrogen inputs, over four sampling years (2018–2021).

It comprises 640 samples for which sequencing data were obtained, and 595 samples after quality control, representing 10,633 operational taxonomic units (OTUs) derived from 16S rRNA gene amplicon sequencing. Available data include raw sequencing reads deposited in a public repository, an OTU count table with taxonomic annotation, a sample metadata table describing experimental design and management variables, and phyloseq objects in R format. Reproducible analysis outputs are also provided as HTML documents describing data structure, quality control procedures, and diversity metrics.

These data provide a structured resource for exploring soil bacterial community composition across contrasting pesticide-free agroecological cropping systems in arable crops. The availability of curated data objects and associated metadata facilitates reuse for methodological developments, benchmarking of bioinformatics workflows, and comparative analyses with other soil microbiome datasets.

Specifications Table

**Table:** 

Subject	Biology
Specific subject area	Soil Metagenomics, Agricultural Microbiology, Agroecological practices, Microbiome, Bioinformatics
Type of data	16S rRNA gene amplicon sequencing data: raw sequencing reads (FASTQ files) and processed data including OTU count table with taxonomic information (.csv), sample metadata (.csv), phyloseq objects (raw and filtered, .rds), and reproducible analysis outputs (HTML vignettes generated in R).
Data collection	Soil samples (*n* = 640) were collected from 42 experimental plots (4 subplots/plot) across four cropping systems (2018–2021) in the CA-SYS platform (France). Soil was sampled at the same location (precision 5 cm, RTK) across years. DNA was extracted from 250 to 300 mg soil using the DNeasy 96 PowerSoil Pro kit (Qiagen). The 16S rRNA gene (V3–V4 region) was amplified using primers Pro341F/Pro805R and sequenced on an Illumina MiSeq platform (2 × 250 bp). Sequence processing generated OTU table. Quality filtering based on sequencing depth and contamination criteria resulted in a final dataset of 595 samples; excluded samples remain available in the raw dataset.
Data source location	Experimental farm U2E of INRAE (CA-SYS platform), Bretenière, France (47°14′11.2″ N, 5°05′56.1″ E)
Data accessibility	**16S rRNA amplicon sequencing (raw reads)** Repository name: The National Center for Biotechnology Information (NCBI) Data identification number: PRJNA1453205 Direct URL to data: https://www.ncbi.nlm.nih.gov/bioproject/PRJNA1453205 **Processed microbiome datase**t **(OTU table, metadata, and reproducible analysis reports)** Repository name: Data INRAE Data identification number: doi:10.57745/LO2KIF Direct URL to data: https://entrepot.recherche.data.gouv.fr/dataset.xhtml?persistentId=doi:10.57745/LO2KIF
Related research article	none

## 1 Value of the Data

• This dataset provides soil bacterial community profiles from a long-term experimental platform comparing four agroecological cropping systems under contrasting soil management practices and fertilisation regime without pesticide use. The CA-SYS platform assess the feasibility and effect of pesticide-free agriculture by testing agroecological strategies moving cropping systems towards less soil disturbance (such as in conservation agriculture) and less reliance on chemical nitrogen fertilisation (such as in organic agriculture).
• Multi-year observations across replicated experimental plots, including a baseline sampling prior to the full establishment of management practices, allow the study of temporal dynamics and variability of soil microbial communities under field conditions.
• Soil samples were collected at the same georeferenced locations across years using RTK precision, minimising spatial confounding between sampling dates and strengthening the comparability of temporal observations.
• The availability of processed data, including curated phyloseq objects, associated metadata, and five reproducible analysis outputs (HTML vignettes in R), allows reuse for method development, benchmarking of bioinformatics pipelines, and comparative analyses.
• The dataset enables the investigation of relationships between soil microbial communities and agricultural practices in a large-scale field experiment, based on a substantial number of samples collected across systems and years.

## 2 Background

CA-SYS is a field experimental platform set up and run by INRAE to evaluate agroecological practices at the field and landscape scale. CA-SYS aims at assessing a variety of agroecological cropping systems across 125 hectares, using either reduced tillage (no-till or superficial tillage) or conventional tillage (ploughing), all without pesticides (neither biopesticides), to assess the benefits of reducing synthetic inputs in favour of enhancing biotic interactions within a fragmented landscape rich in ecological infrastructure (flower strips, grass strips, hedges). The dataset documents soil bacterial diversity and community structure across four agroecological cropping systems. It covers a four-year period (2018–2021), including an initial baseline sampling in 2018 prior to the establishment of the CA-SYS systems, followed by three years of observations (2019–2021) after their implementation.

## 3 Data Description

### 3.1 Data overview

The dataset comprises soil bacterial communities characterised by 16S rRNA gene amplicon sequencing within the CA-SYS experimental platform. The experimental design includes four agroecological cropping systems differing in soil management practices: SD1 (permanent no-till based system), SD2 (non-permanent no-till based system), TS1 (ploughing based system with exogenous nitrogen fertilisation), and TS2 (ploughing based system without exogenous nitrogen fertilisation). These systems are designed to explore agroecological strategies based on reduced soil disturbance, reduced chemical fertilisation and the absence of pesticide use. The SD1 system follows conservation agriculture (CA) principles (sensus [1,2] since SD1 is permanent no-till (no tillage at all over the crop rotation) with cover crop in a diversified crop rotation. The SD2 system is non-permanent no-till (or pseudo-CA, sensus [3]) since it intends to follow CA principles (no-till, cover crop and diversified crop rotation) but allows superficial tillage once a year before sowing to manage pests. All systems are based on diversified crop rotations, with crops grown as pure stands or as intercrops, including companion crops (e.g., oilseed rape with legumes), and with cover crops during the fallow period.

These four cropping systems explored two main agricultural paths on CA-SYS: (i) SD, i.e. systems that tend to minimise or even do without tillage altogether, maximising plant cover, rather inspired by soil conservation agriculture (CA), and (ii) TS, i.e. systems that mobilize tillage (including ploughing, non-inversion deep tillage and superficial tillage) and mechanical weeding, but not in a systematic way, systems rather inspired by organic agriculture. The idea is that these two major paths, which rightly claim to be agroecological, can easily be pushed further apart: conservation agriculture is still dependent on certain pesticides, particularly glyphosate; on the other hand, organic agriculture still relies too much on tillage, which, when too intensive, negatively impact soil health.

**Fig. 1:**
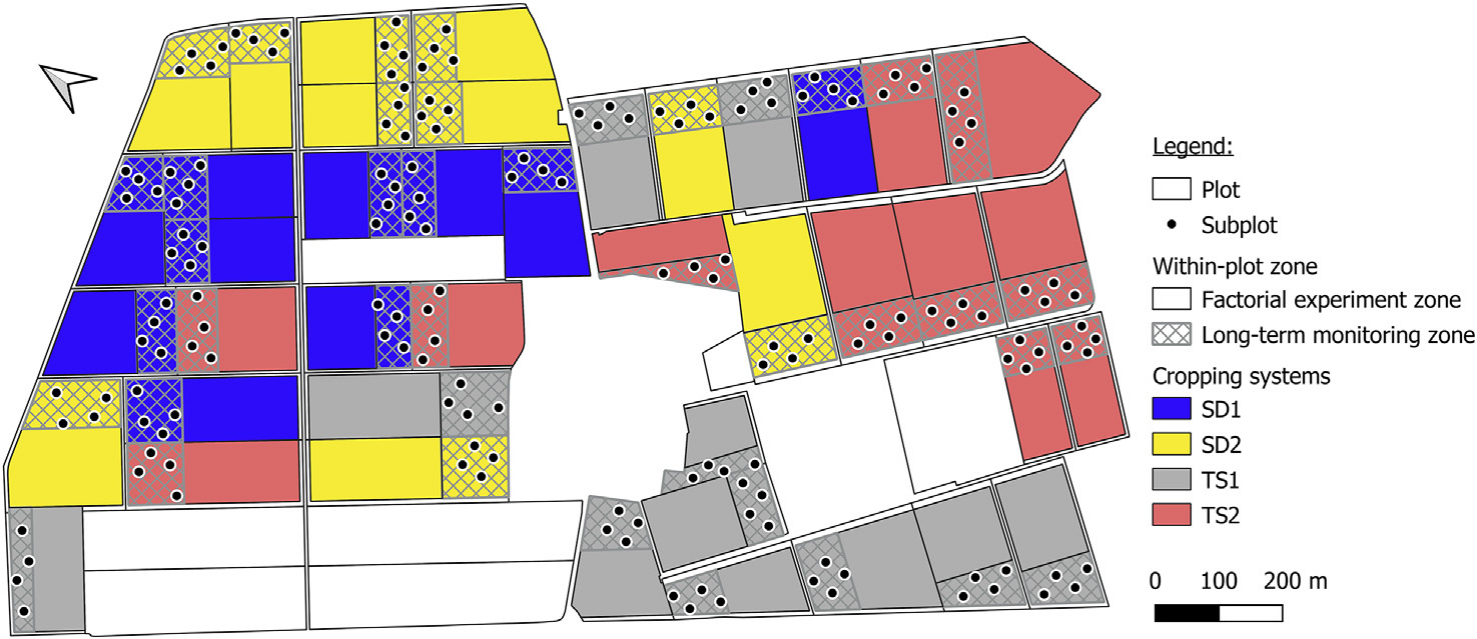
Spatial organisation of the soil sampling and the cropping systems across the CA-SYS experimental platform. Each of the 42 plots are managed by one cropping system (SD1, SD2, TS1, TS2). Each plot is divided into two zones: the factorial experiment zone where analytical and annual experiments are located and the long-term monitoring zone when the soil sampling and all taxa are monitored in the subplot.

Each field was managed under one of these four cropping systems (Fig. 1): TS1 (*n* = 11), TS2 (*n* = 11), SD1 (*n* = 10), and SD2 (*n* = 10). TS systems are tillage-based, while SD systems rely on direct sowing.

TS1 and TS2 both included occasional ploughing (once every three years). TS1 allowed the use of mineral nitrogen fertilisers, whereas TS2 operated without fertilisation, aiming to rely on natural nitrogen fixation and nutrient uptake processes. As a result, practices differ: TS2 included more legumes, more cereal–legume intercrops, and fewer nitrogen-demanding crops. SD1 is a strict no-till system following conservation agriculture principles, while SD2 allowed shallow tillage once a year before sowing (minimum tillage). All systems used diversified crop rotations, including sole crops and intercrops (e.g., oilseed rape with legumes), along with cover crops during fallow periods.

Irrigation was permitted in all systems but remains limited (maximum two applications of 40 mm per year for summer crops). Nitrogen fertilisation is allowed in SD1, SD2, and TS1, with a target yield set at 10% below local potential (e.g., 7 t·ha⁻¹ for winter wheat). Winter wheat and oilseed rape were sown using mixtures of varieties (four and three, respectively) to reduce pest pressure while maintaining yield and quality. No pesticides and biopesticides (including seed treatments) were used in any system.

The dataset follows a hierarchical structure, with samples nested within sampling points, sampling points within plots, and plots distributed across cropping systems and years. A total of 672 soil samples were initially collected (42 plots * 4 subplots/plot) across four sampling years (2018–2021). Samples collected in 2018 corresponded to the initial phase (baseline) of the experimental platform prior to the full establishment of management practices. Plots were farmed under conventional management, i.e. all sprayed with pesticides, under intensive tillage (including ploughing) and fertilised with chemical inputs. All the crops at sampling and the 3 preceding crops were presented in the metadata.

After sequencing and initial processing, 640 samples were retained in the raw dataset, corresponding to the samples for which sequencing data were available. Following quality filtering based on sequencing depth and contamination criteria, 595 samples were included in the filtered dataset used for downstream analyses. Both raw and filtered datasets are provided, allowing users to access the complete set of samples as well as the subset used for downstream analyses.

The dataset comprises 10,633 operational taxonomic units (OTUs), defined as clusters of similar 16S rRNA gene sequences obtained after sequence processing and taxonomic assignment. More than half of the OTUs have low total abundance (<100 reads across all samples), reflecting the presence of a long tail of rare taxa. The OTU table exhibits a sparse structure, with 74.6% of entries equal to zero. A large proportion of OTUs are of low abundance, while a small subset of highly abundant OTUs dominates the dataset, with the top 1% of OTUs accounting for 44.6% of total reads.

The dataset is organised into complementary components distributed across two repositories. Raw sequencing reads (FASTQ files) are available in the NCBI Sequence Read Archive. Processed data are provided in the Data INRAE repository and include an OTU count table, a sample metadata table, two phyloseq objects (raw and filtered), and five reproducible analysis outputs (HTML reports).

Table 1 summarizes these components, including file types, formats, and their associated sections.

**Table 1:** Overview of the dataset structure and associated files.

Data type	Description	File format	File name / location	Associated section
Raw sequencing reads	16S rRNA gene amplicon sequences	FASTQ	NCBI SRA (BioProject PRJNA1453205)	Experimental design, materials and methods
OTU count table	OTU abundance matrix with taxonomic annotation	CSV	casys_otu_table_raw.csv	Data description
Sample metadata	Experimental variables and sample information	CSV	casys_sample_metadata.csv	Data description
Phyloseq object (raw)	Unfiltered dataset including all samples	RDS	casys_phyloseq_raw.rds	Data description
Phyloseq object (filtered)	Dataset after quality filtering	RDS	casys_phyloseq_clean.rds	Quality control
Analysis outputs	Reproducible analysis reports (diversity and composition)	HTML	01_build_phyloseq_object.html; 02_quality_control.html; 03_taxonomic_composition.html; 04_alpha_diversity.html; 05_beta_diversity.html	Data description

The sample metadata table contains descriptive variables associated with each sample, including experimental design variables (e.g., cropping system, plot, year, subplot, with the sampling point corresponding to the centre of each subplot), agricultural management practices (e.g., tillage, fertilisation, crop at the time of soil sampling, bare soil if no crop), and sequencing information (sequencing depth), and sequencing information (sequencing depth). Additional variables include soil type classification and soil resistivity measurements (Resist_V1_cor for the 0–50 cm soil layer, Resist_V2_cor for the 0–100 cm soil layer, Resist_V3_cor for the 0–170 cm soil layer), derived from a previously published dataset [4]. Resist_PC1 is a derived variable corresponding to the first principal component obtained from a principal component analysis (PCA) performed on these resistivity variables. These metadata variables allow the linkage between microbial community data, experimental design and management practices.

### 3.2 Quality control

Sequencing depth was used as a primary criterion for data quality assessment. Samples with fewer than 11,000 reads were classified as low-depth and excluded from the filtered dataset, resulting in a minimum sequencing depth of 11,123 reads across retained samples. This threshold was chosen to ensure sufficient sequencing depth for reliable diversity estimation across samples. In addition, a small number of samples identified as contaminated, based on abnormal taxonomic profiles characterised by the dominance of a single taxon, were removed, resulting in a final dataset of 595 samples. The distribution of samples across cropping systems and years after quality filtering is summarized in Table 2.

**Table 2:** Distribution of samples across cropping systems and years after quality filtering.

System	2018	2019	2020	2021	Total
SD1	32	33	38	38	141
SD2	33	34	38	39	144
TS1	35	36	43	44	158
TS2	32	36	42	42	152
**Total**	132	139	161	163	595

**Fig. 2:**
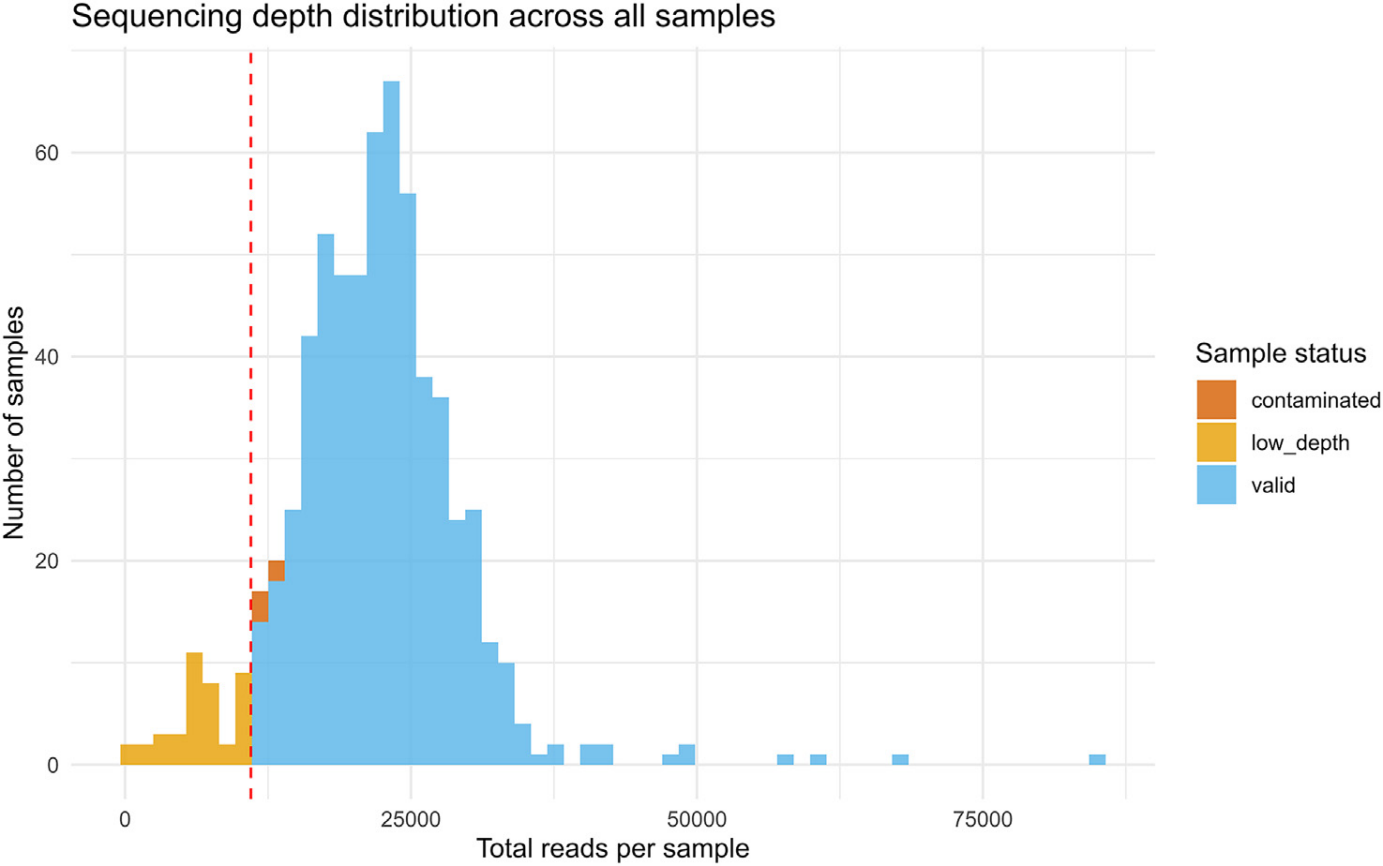
Distribution of sequencing depth across all samples. The dashed line indicates the filtering threshold at 11,000 reads. Samples are coloured according to their status (valid, low-depth, contaminated).

The distribution of sequencing depth across all samples is shown in Fig. 2, with the filtering threshold indicated.

Rarefaction curves were computed to assess sequencing completeness across samples and confirmed that the selected depth threshold was sufficient to capture the majority of diversity.

### 3.3 Taxonomic composition

After sequence processing and filtering, a total of 10,633 OTUs were identified across the dataset. Taxonomic assignment revealed a broad diversity of bacterial taxa, spanning 47 phyla, 140 classes, 341 orders, 535 families, and 1019 genera.

The most abundant phyla across all samples were Actinobacteriota, Proteobacteria, and Acidobacteriota.

**Fig. 3:**
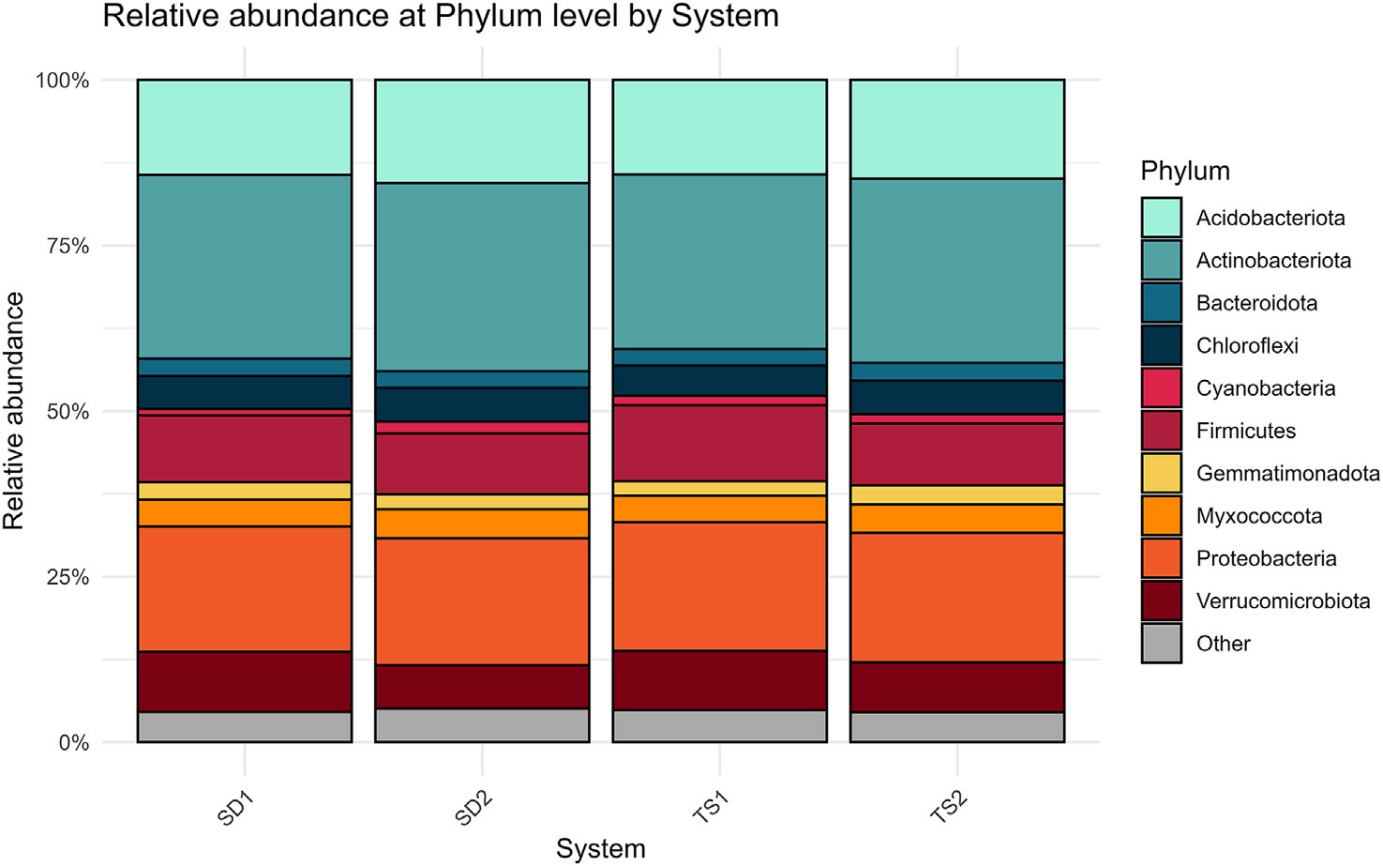
Relative abundance of dominant bacterial phyla across cropping systems. The 10 most abundant phyla are shown, with remaining taxa grouped as “Other”.

Relative abundance profiles at the phylum level across cropping systems are shown in Fig. 3, with the 10 most abundant phyla displayed and remaining taxa grouped as “Other”.

### 3.4 Alpha diversity

Alpha diversity was assessed using Shannon and inverse Simpson indices, calculated after multiple rarefaction (100 iterations) to a sequencing depth of 11,123 reads, corresponding to the minimum sequencing depth across retained samples above the quality filtering threshold (11,000 reads). Diversity indices were computed over 100 rarefaction iterations and averaged across runs.

Differences in alpha diversity across cropping systems and years were evaluated using Kruskal–Wallis tests.

**Fig. 4:**
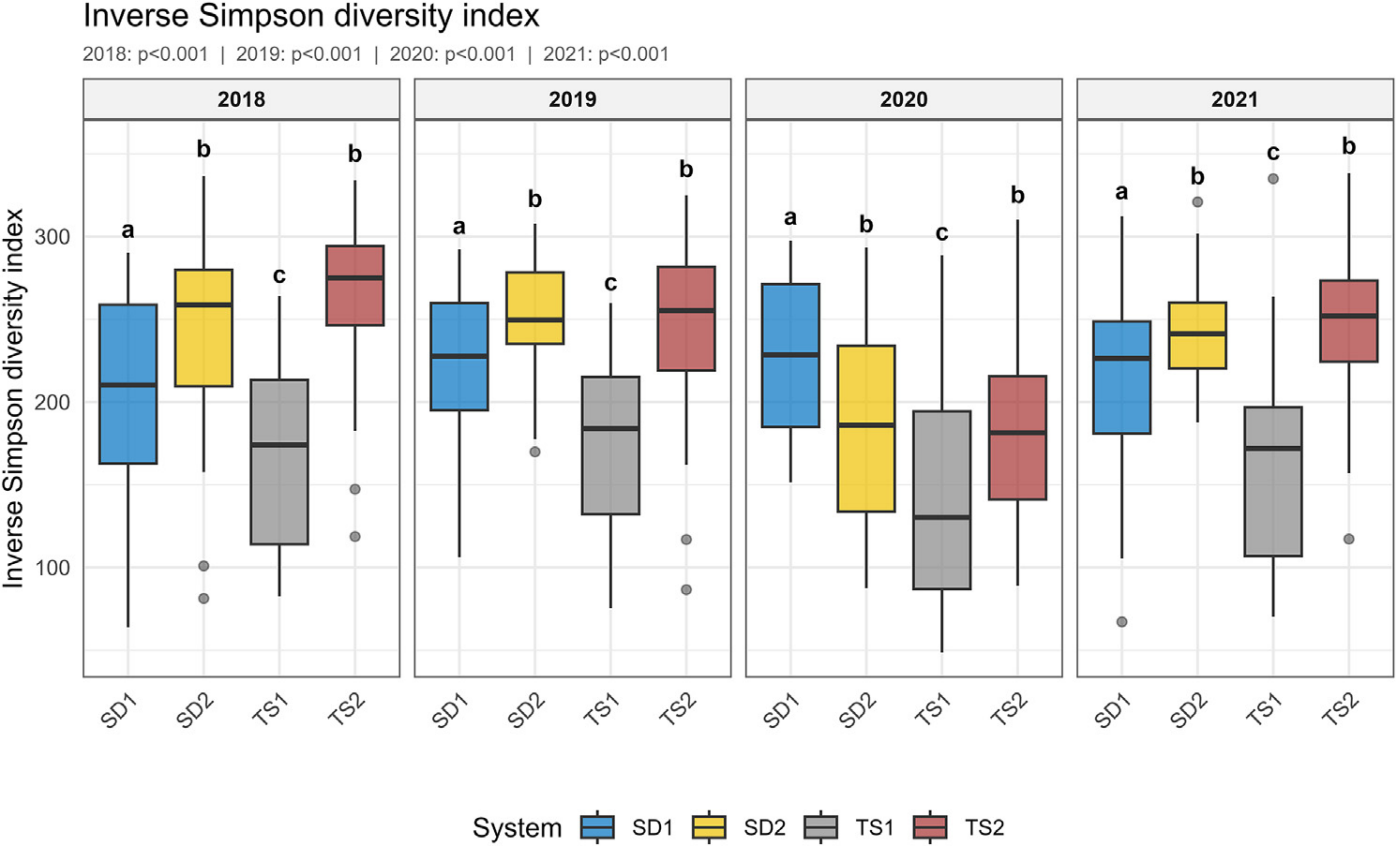
Inverse Simpson diversity index across cropping systems and sampling years. Values are averaged over 100 rarefaction iterations at a depth of 11,123 reads. The boxplot uses compact letter displays (CLD) to indicate significant differences between groups.

Alpha diversity patterns based on the inverse Simpson index are shown in Fig. 4.

### 3.5 Beta diversity

**Fig. 5:**
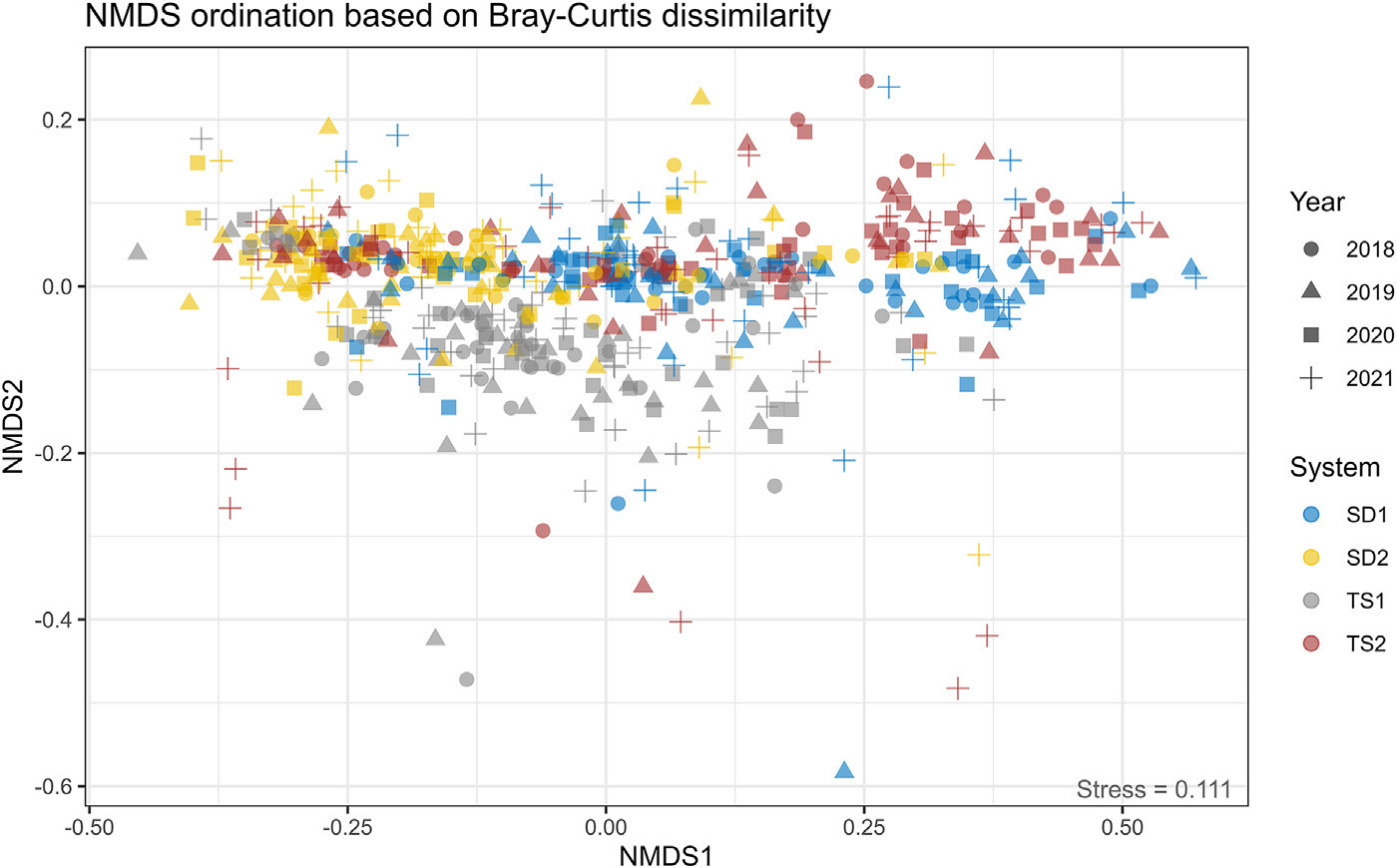
NMDS ordination based on Bray–Curtis dissimilarity of Hellinger-transformed OTU data. Samples are coloured by cropping system and shaped by sampling year. Stress = 0.111 indicates the goodness-of-fit of the ordination.

Beta diversity was assessed using Bray–Curtis dissimilarity computed on Hellinger-transformed OTU data. Community structure was visualised using non-metric multidimensional scaling (NMDS), as shown in Fig. 5.

Differences in community composition across cropping systems and years were evaluated using Permutational Multivariate Analysis of Variance (PERMANOVA).

## 4 Experimental Design, Materials and Methods

### 4.1 Soil sampling

The soils were sampled in the CA-SYS experiment. It is located in Burgundy near Dijon (47°14′10’’N, 5°05′53’’E), characterised by a semi-continental climate, with cold and wet winters, warm summers, and a mean annual precipitation of 761 mm. The soil is 60 to 90 cm deep with a silty-clay texture.

Soil samples were collected from 42 experimental plots (4 subplots/plot) across four cropping systems (2018–2021) in the CA-SYS platform (France). Soil was sampled at the same location (precision 5 cm, RTK) across years. Soil was sampled from 0–25 cm depth with 5 soil cores (5 cm diameter probe) combined to form a representative composite sample of 3–5 kg per subplots depending on the soil texture and humidity. A subsample was processed through the GENOSOL protocol and stored in the GENOSOL platform. Indeed, the soil was then homogenised and sieved (typically at 4 mm) to remove large debris like stones and roots. A subsample was taken from this mixture to ensure it accurately represents the original field conditions. The samples were carefully stored (−40 °C) after a lyophilisation process until further DNA extraction.

The batch consists in 672 soil samples dispatched between four dates (2018, 2019, 2020, 2021), corresponding to 168 samples per year.

### 4.2 DNA sample extraction and dilution

Soil DNA was extracted from 250–300 mg of each soil sample using DNeasy 96 PowerSoil Pro kit (Qiagen, Germany), according to the manufacturer’s protocol. The procedure involved the 3 following main steps: (i) microbial cell lysis by chemical and mechanical (bead beating in a TissueLyser apparatus (Qiagen, Germany)) actions, (ii) chemical deproteination and (iii) DNA precipitation and washing using separation columns.

The final elution was performed in 100 µl of elution buffer (provided in the kit). DNA concentrations were determined using an Infinity Pro 200 device (Tecan, Switzerland), with the Quant-iT™ PicoGreen® dsDNA assay kit (Invitrogen, USA), a quantification method based on a fluorescent dye, Picogreen®. The quantified DNA samples were diluted at a final concentration of 1 ng/µl using a pipetting robot (Tecan, Switzerland).

### 4.3 16S rDNA libraries preparation

Briefly, the PCR reaction mixture consisted, for each sample, of Phusion High Fidelity Master Mix (Thermo Fisher, France) at a final concentration (FC) of 1X, primers Pro341F 5′-TCGTCGGCAGCGTCAGATGTGTATAAGAGACAGNNNNCCTACGGGNBGCASCAG-3′ / Pro805R 5′-GTCTCGTGGGCTCGGAGATGTGTATAAGAGACAGNNNNGACTACNVGGGTATCTAATCC-3′ at a FC of 1 µM, 0.05 µl of T4 gp32 (MP Biomedicals, France), and ultrapure “molecular biology grade” water completing a final reaction volume of 15 µl. For each sample, 2 µl of extracted DNA diluted to 1 ng/µl (i.e. 2 ng) were added to this mixture.

First step PCR reactions were performed in a SimpliAmp thermocycler (Life Technologies SAS, USA) using the following program: an enzymatic activation step at 98 °C for 3 min, followed by the amplification steps consisting of 25 cycles under the following conditions: 98 °C for 30 s, 55 °C for 30 s, 72 °C for 30 s, and ultimately a final extension step at 72 °C for 10 min. This 1st step PCR was done in duplicate, and the two replicates were then pooled to a final volume of 30 µl.

A second PCR was then performed to bind tag sequences to the 16S amplicons. The reaction mixture consisted of Phusion High Fidelity Master Mix (Thermo Fisher, France) at FC 1X and ultrapure “molecular biology grade” water to reach a final volume of 30 µl. Each reaction also received a unique pair of tag sequences at a final concentration of 1 µM each. The PCR template consisted of 6 µl of 1st step PCR product. 2nd step PCR reactions were performed in a SimpliAmp thermocycler (Life Technologies SAS, USA) using the following program: an enzyme activation step at 98 °C for 3 min, followed by the amplification steps consisting of 8 cycles under the following conditions: 98 °C for 30 s, 55 °C for 30 s, 72 °C for 30 s, and at last a final extension step at 72 °C for 10 min. This 2nd step PCR were done in duplicate, and the two replicates were then pooled to obtain a final volume of 60 µl.

Second step PCR products were then purified and normalised using SequalPrep Normalisation Plate Kit (Applied Biosystems, USA) according to the manufacturer’s protocol. The 96-well plates of normalised samples were stored at −20 °C until shipment to an external private company, Genoscreen (Lille, France), after all samples have been pooled into a 2 ml tube, for MiSeq sequencing (2 × 250 bp).

### 4.4 Bioinformatics and statistical analyses

Bioinformatics processing and statistical analyses were performed in R (version 4.5.2) [5]. Sequence processing and OTU table construction were carried out using in-house developed Python pipeline (https://forge.inrae.fr/vasa/illuminametabarcoding). 16S sequences were assembled with PEAR [6] with default settings. Further quality checks were performed to remove short sequences (<400 bp). Reference-based and de novo chimera detection, as well as OTU clustering were performed using VSEARCH [7] and the SILVA [8] representative set of sequences. Representative sequences for each OTU were aligned using INFERNAL [9] and a phylogenetic tree built using FASTTREE [10]. Finally, taxonomy was assigned using BLAST and the SILVA database (138.1/2020). OTU tables and sample metadata were integrated into phyloseq objects using the phyloseq R package (v1.54.0) [11]. Quality filtering was applied based on sequencing depth and contamination criteria to generate the filtered dataset used for downstream analyses. Samples with fewer than 11,000 reads were classified as low-depth and excluded. Samples identified as contaminated were removed taxonomic criteria, as described in the Quality Control section. Data analysis and visualization were performed using the ggplot2 R package (v4.0.1) [12].

Alpha diversity indices (Shannon and inverse Simpson) were calculated after multiple rarefaction (100 iterations) to a standardised sequencing depth of 11,123. Differences in alpha diversity across cropping systems within each year were assessed using Kruskal–Wallis tests, followed by pairwise Wilcoxon tests with Benjamini–Hochberg false discovery rate correction. Statistical significance was assessed at a threshold of *p* < 0.05.

Beta diversity was assessed using Bray–Curtis dissimilarity on Hellinger-transformed OTU data. Community structure was explored using non-metric multidimensional scaling (NMDS). Differences between groups were assessed using PERMANOVA and pairwise PERMANOVA, implemented using the vegan (v2.8–0) [13] and pairwiseAdonis (v0.4.1) [14] packages.

## Limitations

The experimental design is structured such that each plot is associated with a single cropping system throughout the duration of the study. Consequently, cropping system effects may be partially confounded with plot-specific characteristics, including initial soil conditions. However, the spatial heterogeneity of the soil is well characterised and provided in the database through several variables allowing to partially disentangle the effect of cropping systems from soil parameters.

Differences were observed for samples collected in 2018 compared to subsequent years, particularly in sequencing depth and observed richness. These differences may reflect either biological variability or technical variation related to sequencing conditions, which need to be carefully dealt with in the statistical analyses.

A subset of samples was excluded during quality filtering due to low sequencing depth or contamination. These exclusions are not uniformly distributed across plots and years, which may introduce minor imbalances in the dataset.

Finally, the dataset spans four sampling years (2018–2021), which may limit the assessment of longer-term temporal dynamics.

## Ethics Statement

The authors have read and follow the ethical requirements for publication in Data in Brief and confirm that the current work does not involve human subjects, animal experiments, or any data collected from social media platforms.

## CRediT Author Statement

**Elizaveta Klockenbring:** Formal analysis; Methodology; Writing - Original Draft; **Julie Aubert:** Validation; Methodology; **Jérémie Béguet:** Software; Writing - Original Draft; **Stephane Cordeau:** Project administration ; Data Curation; Writing - Review & Editing; **Violaine Deytieux:** Project administration ; Data Curation; **Céline Faivre:** Data Curation; Writing - Review & Editing; **Rodolphe Hugard:** Data Curation; **Nicolas Jouvin:** Validation; Methodology; **Samuel Mondy:** Resources; **Brice Mosa:** Data Curation; **Aymé Spor:** Supervision; Writing - Review & Editing.

## Data Availability

data INRAE (Recherche Data Gouv)16S rRNA gene sequencing data of soil bacterial communities from four agroecological cropping systems in the CA-SYS experiment (2018-2021) (Original data). NCBI Sequence Read Archive (SRA)16S rRNA gene sequencing data of soil bacterial communities from four agroecological cropping systems in the CA-SYS experiment (2018-2021) (Original data).
